# Corvis ST parameters in primary open angle and angle closure glaucoma after propensity score matching

**DOI:** 10.1038/s41598-026-57462-w

**Published:** 2026-06-11

**Authors:** Yuta Nakaniida, Kana Tokumo, Shunsuke Nakakura, Ryo Asaoka, Yoshiaki Kiuchi, Hirokazu Sakaguchi

**Affiliations:** 1 Department of Ophthalmology and Visual Science, Hiroshima University, 1-2-3 Kasumi, Minami-ku, Hiroshima, 734-8551 Japan; 2 Department of Ophthalmology, Saneikai Tsukazaki Hospital, 68-1 Aboshi Waku, Himeji, 671-1227 Japan; 3 Department of Ophthalmology, Seirei Hamamatsu General Hospital, 2-12-12 Sumiyoshi, Naka-ku, Hamamatsu City, Shizuoka 430-8558 Japan

**Keywords:** Corvis ST, Primary open-angle glaucoma, Primary angle-closure glaucoma, Corneal biomechanics, Intraocular pressure, Diseases, Medical research

## Abstract

This retrospective study evaluated differences in Corvis ST tonometry (CST) parameters between eyes with primary open-angle glaucoma (POAG) and primary angle-closure glaucoma (PACG) using propensity score matching. We analyzed 48 eyes with POAG and 48 eyes with PACG. Twelve CST parameters were compared using linear mixed-effects models, with group as a fixed effect and subject ID as a random intercept to account for inter-eye correlation, adjusting for age, sex, axial length, intraocular pressure, and central corneal thickness. Multiple comparisons were controlled using the Benjamini–Hochberg procedure. Compared with POAG eyes, PACG eyes showed longer A2 time, greater A2 deformation amplitude, and greater whole-eye movement; however, these differences were no longer statistically significant after correction for multiple testing. After adjustment for relevant covariates and correction for multiple comparisons, this study did not demonstrate statistically significant differences in CST parameters between POAG and PACG eyes. The uncorrected trends toward greater indentation-related responses in PACG eyes should be regarded as exploratory.

## Introduction

Glaucoma is a leading cause of blindness worldwide and is characterized by irreversible visual impairment^1^. Primary open-angle glaucoma (POAG) and primary angle-closure glaucoma (PACG) differ in their pathophysiology and ocular morphological features^2^.

The Corvis ST (corneal visualization Scheimpflug technology) is a noncontact device that delivers an air puff to the cornea and captures the deformation response using a high-speed Scheimpflug camera, enabling the quantitative assessment of corneal and whole-eye biomechanical properties^3^.

Corvis ST (CST) parameters are influenced by several factors, including age, sex, axial length (AL), intraocular pressure (IOP), and central corneal thickness (CCT)^2,4–8^. Therefore, potential confounders must be carefully addressed when examining their associations with glaucoma pathophysiology.

In our previous comparison of POAG and PACG eyes, we identified significant differences in five CST parameters^2^. However, baseline characteristics were imbalanced: the PACG group included a higher proportion of women and had a significantly shorter AL. Notably, all five parameters were also significantly correlated with AL, raising uncertainty as to whether the observed differences reflected true pathophysiological variation or were attributable to baseline factors such as AL.

Herein, we applied propensity score matching based on age, sex, AL, IOP, and CCT, followed by linear mixed-effects model (LMM) analyses adjusted for these variables. We also implemented correction for multiple comparisons to limit false-positive findings.

Our objective was to clarify whether differences in CST parameters between POAG and PACG reflect underlying pathophysiological variation while minimizing the influence of potential confounders.

## Materials and methods

This retrospective cross-sectional study included 48 eyes with POAG and 48 eyes with PACG. Data were collected at Hiroshima University Hospital, Saneikai Tsukazaki Hospital, and Seirei Hamamatsu General Hospital between August 1, 2019, and December 25, 2021.

POAG and PACG were diagnosed according to the Japan Glaucoma Society Guidelines for Glaucoma (fifth edition)^9^. POAG was defined as (1) typical glaucomatous optic nerve head changes, including a rim notch with rim width ≤ 0.1 disc diameters or a vertical cup-to-disc ratio of > 0.7, and/or a retinal nerve fiber layer defect at the optic nerve head margin wider than a major retinal vessel, diverging in an arcuate or wedge shape and (2) a wide open angle on gonioscopy. POAG eyes with significant cataract were excluded, except for clinically insignificant senile cataracts detected on biomicroscopy.

Eyes were classified as having PACG when both of the following criteria were met: (1) gonioscopic angle closure, defined as an inability to visualize the posterior pigmented trabecular meshwork over ≥ 180° in primary gaze without indentation, and (2) glaucomatous optic neuropathy, indicated by neuroretinal rim thinning with a vertical cup-to-disc ratio of > 0.7, inter-eye vertical cup-to-disc ratio asymmetry of > 0.2, and/or focal rim notching, accompanied by a corresponding glaucomatous visual field defect. PACG eyes were referred from other hospitals for laser or surgical treatment following drug therapy, and all examinations were performed before these interventions. When both eyes of a participant met the eligibility criteria, data from both eyes were included in the analysis.

Eyes with a history of cataract surgery or pseudophakia were excluded. All included eyes were phakic.

### Ethics approval

The study protocol was approved by the Ethical Committee for Epidemiology of Hiroshima University (permission No. Epd-826-4). Written informed consent was obtained from all participants for storage of clinical data in the hospital database and their use for research purposes. The study adhered to the tenets of the Declaration of Helsinki.

This study involved only in vivo clinical measurements in living participants and did not involve human ocular tissues or cells (e.g., postmortem donor tissue or cultured cells). Accordingly, donor-related information such as cause of death, death-to-preservation interval, and ventilator time was not applicable.

### CST measurement

The operating principle of the CST has been described previously^3^. Briefly, a high-speed Scheimpflug camera captures a sequence of corneal images during the cornea’s dynamic response to an air-puff stimulus. Images are acquired at 4,330 frames per second, and the data are processed to derive parameters such as CCT, deformation amplitude, length of the deformed corneal region, and corneal motion velocity.

CST parameters were defined as follows. A1 time is the interval from air-puff onset to the first applanation, representing initial corneal flattening during inward deformation. In noncontact tonometry, IOP can be estimated from A1 time^6,10–12^. A2 time corresponds to the second applanation, occurring as the cornea rebounds outward toward its baseline shape after initial inward deformation; following the first applanation, the cornea continues posterior movement before the recovery phase begins. The velocity of the corneal apex at A1 and A2 times is defined as A1 and A2 velocity, respectively, while the length of the flattened cornea at these time points is defined as A1 and A2 length, respectively. The magnitude of corneal deformation at A1 and A2 times was defined as A1 and A2 deformation amplitude, respectively. At the highest concavity (HC), deformation amplitude represents the maximum corneal deformation. Peak distance is the separation between the two peripheral deformation peaks observed at HC, and radius refers to the radius of curvature of the central cornea at HC. Whole-eye movement is defined as the maximum posterior displacement of the entire eye induced by the air-puff stimulus.

Corvis ST examinations (software v1.6r2031) were performed three times per eye on the same day, with a minimum 1-min interval between measurements. The mean of the three readings was used for each parameter, and only acquisitions that passed the instrument’s on-screen quality check (quality index: “OK”) were included in the analysis.

### Other measurements

AL was measured using IOLMaster 700 (Carl Zeiss Meditec, Dublin, CA, USA), IOP was determined by Goldmann applanation tonometry, and CCT was measured with the CST.

### Sequence of measurements

All examinations were performed on the same day. Because of the retrospective multicenter design, the exact sequence of CST examination, Goldmann applanation tonometry, and ocular biometry was not uniformly standardized.

### Statistical analysis

To minimize baseline imbalances between groups, we performed propensity score matching^13,14^ at the eye level, because CST parameters and ocular covariates were measured for each eye. A propensity score represents the estimated probability of belonging to the PACG group conditional on observed baseline covariates. Propensity scores were estimated using a logistic regression model including age, sex, IOP, AL, and CCT as covariates. We applied 1:1 nearest-neighbor matching without replacement on the logit of the propensity score. The caliper width was set to 0.2 times the standard deviation of the logit of the propensity score, corresponding to a raw caliper width of 0.2^14^ in this dataset. The random seed was set to 20,260,101 for reproducibility. From 144 potentially eligible POAG eyes, 48 were matched 1:1 to 48 PACG eyes selected from 92 potentially eligible PACG eyes.

Covariate balance before and after propensity score matching was assessed using standardized mean differences (SMDs) and visualized using a Love plot. The SMD quantifies the magnitude of between-group differences in each covariate on a standardized scale and is less affected by sample size than P-values. For continuous variables, SMDs were calculated as the difference in means divided by the pooled standard deviation, whereas for categorical variables, they were calculated as the difference in proportions standardized by the pooled binomial variance. An absolute SMD of < 0.10 was considered a target for negligible imbalance, whereas larger values suggested residual imbalance. A Love plot displays the absolute SMDs for each covariate before and after matching, with values closer to zero indicating better covariate balance. The term “distance” refers to the propensity score distance derived from the estimated propensity score used for matching. Inter-eye correlation after matching was accounted for in subsequent linear mixed-effects models by including subject ID as a random intercept.

Effect sizes were reported as adjusted mean differences between groups, calculated as the fixed-effect estimate for group, PACG minus POAG, from the adjusted LMMs. Corresponding 95% confidence intervals were calculated for each adjusted difference.

Continuous variables are presented as mean (standard deviation) [range] and categorical variables as counts and percentages. At the subject level, age differences were assessed using the Wilcoxon rank-sum test^15^, and sex distribution was compared with Fisher’s exact test^16^.

AL, IOP, CCT, and 12 CST parameters were compared at the eye level using LMMs, with group as a fixed effect and subject ID as a random intercept (1|ID) to account for inter-eye correlation. LMMs extend ordinary linear models by incorporating both fixed and random effects, enabling valid inference in clustered or repeated measurements^17,18^. In ophthalmic studies including both eyes from the same subject, eye-level observations are correlated, violating the independence assumption of ordinary linear regression. Incorporating a subject-specific random intercept accounts for this correlation and prevents underestimation of standard errors^19,20^. AL, IOP, and CCT were analyzed using LMMs as follows:

1 $${\mathrm{Outcome}}\left( {{\mathrm{AL}},{\mathrm{IOP}},{\mathrm{or}}\;{\mathrm{CCT}}} \right)\sim{\mathrm{Group}} + \left( {1|{\mathrm{Subject}}\;{\mathrm{ID}}} \right)$$

To account for potential confounding, adjusted LMMs were fitted with clinically relevant covariates as fixed effects: age, sex, AL, IOP, and CCT. The 12 CST parameters were analyzed using the following model:

2 $${\mathrm{CST}}\;{\mathrm{parameter}}\sim{\mathrm{Group}} + {\mathrm{Age}} + {\mathrm{Sex}} + {\mathrm{AL}} + {\mathrm{IOP}} + {\mathrm{CCT}} + \left( {1|{\mathrm{Subject}}\;{\mathrm{ID}}} \right)$$

Models were implemented in R using the lme4 package^21^, with degrees of freedom and P-values estimated via Satterthwaite’s approximation using the lmerTest package^22,23^.

For multiple comparisons across demographic variables and CST parameters, P-values for the group effect were adjusted using the Benjamini–Hochberg procedure to control the false discovery rate (FDR)^24^.

All analyses were performed in R (version 4.5.2; R Foundation for Statistical Computing, Vienna, Austria). Two-sided P-values < 0.05 were considered statistically significant, and for analyses involving multiple testing, FDR-adjusted Q-values < 0.05 were used.

## Results

Table 1 shows SMDs for the matching covariates before and after propensity score matching. After propensity score matching, the SMDs were substantially reduced for the propensity score distance (1.79 to 0.19), age (0.66 to 0.07), sex (0.41 to 0.04), IOP (0.35 to 0.06), and CCT (0.14 to 0.05), indicating improved balance for these variables. However, residual imbalance remained for AL (0.19). These findings suggest that propensity score matching improved covariate balance overall, although balance was not fully achieved for all matched covariates.

**Table 1:** Standardized mean difference (SMD) before and after propensity score matching

Parameters	Before matching SMD	After matching SMD	
distance	1.79	0.19	
Age (years)	0.66	0.07	
Sex (% women)	0.41	0.04	
AL (mm)	1.69	0.19	
IOP (mmHg)	0.35	0.06	
CCT (µm)	0.14	0.05	

Absolute SMD values closer to 0 indicate better covariate balance between groups; an absolute SMD < 0.1 was considered to indicate negligible imbalance.

“distance” represents the propensity score distance derived from the estimated propensity score.

SMD, standardized mean difference.

**Fig. 1:**
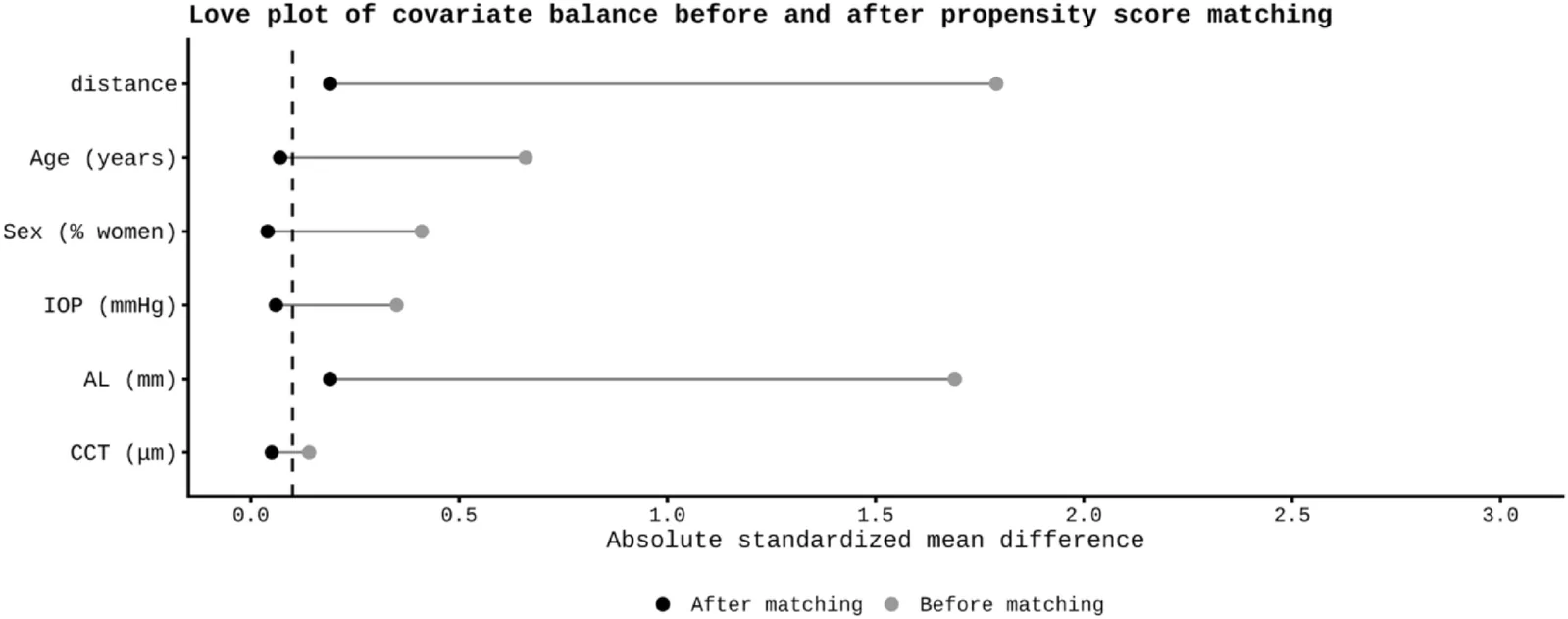
Love plot of covariate balance before and after propensity score matching.

Figure 1 shows the Love plot of covariate balance before and after propensity score matching. Overall, absolute SMDs were reduced after matching, indicating improved balance between groups. After matching, residual imbalance remained for the propensity score distance and AL, both of which had absolute SMDs of 0.19, whereas age, sex, IOP, and CCT had absolute SMDs below 0.10.

Absolute SMDs for the covariates used in propensity score matching are shown before and after matching. Smaller absolute SMD values indicate better covariate balance between groups. The dashed vertical line indicates an absolute SMD of 0.1, a commonly used threshold for negligible imbalance. “distance” represents the propensity score distance derived from the estimated propensity score.

Demographic characteristics of eyes with POAG and PACG are summarized in Table 2. No significant differences were observed in age, sex, AL, IOP, or CCT (*P* = 0.318, 0.618, 0.101, 0.614, and 0.679; Q = 0.679, 0.679, 0.507, 0.679, and 0.679, respectively).

**Table 2:** Demographic characteristics of eyes with POAG and PACG.

Parameters	POAG	PACG	Difference, PACG - POAG	95% CI	*P*-value		Q-value
Subjects	32	35	–	–	–		-
Eyes	48	48	–	–	–		–
Phakic eyes (%)	48/48 (100%)	48/48 (100%)	–	–	–		–
Age (years)	70.81 (9.92) [45.00–81.00]	73.03 (10.45) [49.00–95.00]	2.00	-2.00–6.00	0.318		0.679
Sex (% women)	20 / 32 (62.5%)	24 / 35 (68.6%)	6.1%	-16.7%–28.8%	0.618		0.679
AL (mm)	23.73 (1.05) [21.89–26.71]	23.54 (0.95) [21.84–26.52]	-0.41	-0.90–0.08	0.101		0.507
IOP (mmHg)	16.88 (5.48) [7.00–32.00]	16.25 (4.85) [8.00–35.00]	-0.59	-2.89–1.72	0.614		0.679
CCT (µm)	537.06 (31.62) [442.00–594.00]	536.84 (35.82) [438.00–620.00]	3.40	-12.98–19.79	0.679		0.679

Data are presented as mean (standard deviation) [range] unless otherwise indicated. The reported difference is PACG minus POAG. For AL, IOP, and CCT, differences and 95% confidence intervals were estimated from LMMs. For age and sex, differences and 95% confidence intervals were estimated from the corresponding subject-level analyses.

P-values were calculated using subject-level tests for subject-level variables and eye-level LMMs for eye-level variables. Age was compared using the Wilcoxon rank-sum test, and sex distribution was assessed with Fisher’s exact test. AL, IOP, and CCT were analyzed using LMMs with group as a fixed effect and subject ID as a random intercept (1|ID) to account for inter-eye correlation. Q-values were derived using the Benjamini–Hochberg FDR adjustment across all variables in the table. POAG, primary open-angle glaucoma; PACG, primary angle-closure glaucoma; 95% CI, 95% confidence intervals; AL, axial length; IOP, intraocular pressure; CCT, central corneal thickness.

Comparisons of CST parameters between POAG and PACG eyes are presented in Table 3. PACG eyes showed longer A2 time (*P* = 0.013, Q = 0.099), greater A2 deformation amplitude (*P* = 0.025, Q = 0.099), and greater whole-eye movement (*P* = 0.019, Q = 0.099) compared with POAG eyes; however, these differences were no longer statistically significant after correction for multiple comparisons using the Benjamini–Hochberg method. No significant differences were observed in A1 time, A1 velocity, A1 length, A1 deformation amplitude, A2 velocity, A2 length, HC deformation amplitude, peak distance, or radius between the groups (*P* = 0.059, 0.913, 0.233, 0.597, 0.724, 0.116, 0.059, 0.293, and 0.498; Q = 0.143, 0.913, 0.399, 0.717, 0.789, 0.232, 0.143, 0.439, and 0.664, respectively).

**Table 3:** Comparison of 12 CST parameters between POAG and PACG eyes.

Parameters	POAG	PACG	Adjusted mean difference, PACG - POAG	95% CI	*P*-value	Q-value
A1 time (ms)	7.58 (0.62) [6.87–9.72]	7.40 (0.45) [6.647–9.500]	-0.13	-0.27–0.01	0.059	0.143
A1 velocity (m/s)	0.15 (0.03) [0.09–0.19]	0.15 (0.02) [0.08–0.19]	0.00	-0.01–0.01	0.913	0.913
A1 length (mm)	2.33 (0.22) [1.72–2.82]	2.37 (0.15) [2.15–2.75]	0.04	-0.03–0.12	0.233	0.399
A1 deformation amplitude (mm)	0.14 (0.01) [0.11–0.17]	0.14 (0.01) [0.12–0.17]	0.00	-0.01–0.00	0.597	0.717
A2 time (ms)	21.45 (0.65) [19.96–22.54]	21.74 (0.61) [19.45–22.73]	0.26	0.06–0.46	0.013	0.099
A2 velocity (m/s)	0.26 (0.04) [0.14–0.33]	0.26 (0.04) [0.15–0.32]	0.00	-0.01–0.02	0.724	0.789
A2 length (mm)	3.09 (0.67) [2.22–4.86]	2.92 (0.52) [1.950–4.05]	-0.19	-0.42–0.05	0.116	0.232
A2 deformation amplitude (mm)	0.47 (0.09) [0.24–0.61]	0.52 (0.09) [0.35–0.77]	0.05	0.01–0.09	0.025	0.099
HC deformation amplitude (mm)	1.07 (0.15) [0.60–1.30]	1.11 (0.11) [0.73–1.46]	0.04	0.00–0.07	0.059	0.143
Peak distance (mm)	4.76 (0.43) [3.39–5.39]	4.85 (0.38) [3.69–5.55]	0.07	-0.06–0.20	0.293	0.439
Radius (mm)	6.82 (0.67) [4.84–7.90]	6.85 (0.86) [5.16–8.48]	0.11	-0.21–0.42	0.498	0.664
Whole-eye movement (mm)	0.37 (0.09) [0.13–0.54]	0.42 (0.11) [0.22–0.74]	0.05	0.01–0.09	0.019	0.099

Data are presented as mean (standard deviation) [range]. Adjusted mean differences and 95% confidence intervals were estimated from LMMs adjusted for age, sex, AL, IOP, and CCT.

P-values were calculated using eye-level LMMs, with group as a fixed effect and subject ID as a random intercept (1|ID) to account for inter-eye correlation. Q-values were derived using the Benjamini–Hochberg FDR adjustment across all parameters in the table. POAG, primary open-angle glaucoma; PACG, primary angle-closure glaucoma; 95% CI, 95% confidence intervals; A1, first applanation; A2, second applanation; HC, highest concavity.

## Discussion

In this study, we compared 12 CST parameters between propensity score-matched POAG and PACG eyes using LMMs adjusted for age, AL, sex, IOP, and CCT. Three parameters—A2 time, A2 deformation amplitude, and whole-eye movement—showed statistically significant differences before correction for multiple comparisons. However, after adjustment using the Benjamini–Hochberg procedure, none remained statistically significant. These findings suggest a potential trend toward biomechanical differences between PACG and POAG eyes, but they do not support definitive conclusions regarding glaucoma subtype-specific corneal biomechanics. Nevertheless, the study provides valuable insights by evaluating CST parameters while accounting for major confounders and controlling for multiple testing.

Among the three CST parameters that showed uncorrected between-group differences, A2 time and A2 deformation amplitude are related to the corneal recovery phase after the initial inward deformation. A longer A2 time may indicate delayed return of the cornea toward its baseline configuration, whereas a greater A2 deformation amplitude may reflect a greater deformation response during the second applanation phase. Whole-eye movement represents the maximum posterior displacement of the entire eye induced by the air-puff stimulus and may reflect not only corneal properties but also whole-globe and periocular tissue responses, including ocular rigidity, scleral biomechanics, orbital tissue compliance, and damping of the air-puff-induced displacement. Taken together, the uncorrected trends toward longer A2 time, greater A2 deformation amplitude, and greater whole-eye movement in PACG eyes may suggest greater indentation-related and recovery-phase responses. However, because these findings did not remain statistically significant after FDR correction and because PACG-related anatomical variables were not available, these observations should be interpreted cautiously as exploratory findings.

CST parameters are influenced by age, sex, AL, IOP, and CCT. Because PACG and POAG differ in anatomical characteristics—such as female predominance and shorter AL—simple between-group comparisons are highly susceptible to confounding^2,4–7^. In our previous report, PACG eyes showed longer A2 time, deeper A2 deformation amplitude, shorter peak distance, greater whole-eye movement, and longer whole-eye movement time compared with POAG eyes^2^. Notably, all five parameters were also significantly correlated with AL: peak distance showed a positive correlation, whereas A2 time, A2 deformation amplitude, whole-eye movement, and whole-eye movement time showed negative correlations.

Previous studies comparing CST parameters between POAG and PACG have reported that deformation amplitude and corneal stiffness, calculated using biomechanical models, are significantly higher in PACG and are associated with shallower anterior chamber depth (ACD)^25^. PACG has also been reported to exhibit shallower ACD, thicker CCT, larger corneal radius, and lower maximum inverse radius/integrated radius compared with POAG^26^.

In the present study, we minimized these confounding influences using propensity score matching and covariate adjustment. The loss of statistical significance after correction for multiple comparisons suggests that some observed differences may reflect chance or the impact of testing multiple parameters.

Although PACG eyes showed uncorrected trends toward greater indentation-related responses, these findings did not remain statistically significant after FDR correction. Therefore, these observations should be interpreted as exploratory and do not establish definitive subtype-specific biomechanical differences between POAG and PACG eyes.

In addition to CST parameters, corneal hysteresis (CH), measured using the Ocular Response Analyzer (Reichert Ophthalmic Instruments, Buffalo, NY, USA), is another metric of corneal biomechanics^27,28^. CH reflects corneal viscoelasticity, particularly viscous damping capacity^29^. Eyes with low CH are thought to be mechanically more vulnerable, dissipate less energy, and deform more readily in response to external forces. Studies comparing CH between POAG and PACG have generally found no significant differences, suggesting that subtype-specific biomechanical variations may not be fully captured by CH alone^30^.

This study has several limitations. First, the sample size was relatively small, and statistical power after covariate adjustment and multiple-comparison correction may have been insufficient. Second, the retrospective design may have introduced selection bias. In particular, PACG eyes were referred for laser or surgical treatment, which may not fully represent the broader PACG population. Third, refractive error and spherical equivalent data were not uniformly available and therefore could not be included in the analysis. Fourth, quantitative glaucoma severity indices, including visual field mean deviation, pattern standard deviation, visual field index, and average retinal nerve fiber layer thickness, were not uniformly available at the time of CST measurement. Therefore, glaucoma severity could not be compared between groups or included in the matching or multivariable models. Fifth, PACG-related anatomical parameters, including anterior chamber depth, lens vault, lens thickness, and detailed angle configuration parameters, were not uniformly available. These factors may influence corneal and whole-eye biomechanical responses; therefore, residual confounding related to anterior segment anatomy cannot be excluded. Sixth, detailed information regarding topical antiglaucoma medications, including the number of medications, drug class, duration of treatment, and preservative exposure, was unavailable. Because chronic topical therapy may influence corneal thickness and biomechanical properties^31,32^, medication-related residual confounding cannot be excluded. Future prospective studies with standardized visual field, OCT, anterior segment imaging, and medication data are warranted.

In conclusion, after accounting for age, sex, AL, IOP, and CCT, no statistically significant differences in CST parameters were observed between POAG and PACG eyes. Although several uncorrected trends were observed, no CST parameter remained statistically significant after correction for multiple comparisons. Therefore, the observed tendency toward greater indentation-related responses in PACG eyes should be interpreted as exploratory and hypothesis-generating.

## Data Availability

The dataset for this study is available upon reasonable request to the corresponding author.
